# Volumetric Verification of Multiaxis Machine Tool Using Laser Tracker

**DOI:** 10.1155/2014/959510

**Published:** 2014-06-19

**Authors:** Sergio Aguado, David Samper, Jorge Santolaria, Juan José Aguilar

**Affiliations:** Department of Design and Manufacturing Engineering, University of Zaragoza, Marìa de Luna 3, 50018 Zaragoza, Spain

## Abstract

This paper aims to present a method of volumetric verification in machine tools with linear and rotary axes using a laser tracker. Beyond a method for a particular machine, it presents a methodology that can be used in any machine type. Along this paper, the schema and kinematic model of a machine with three axes of movement, two linear and one rotational axes, including the measurement system and the nominal rotation matrix of the rotational axis are presented. Using this, the machine tool volumetric error is obtained and nonlinear optimization techniques are employed to improve the accuracy of the machine tool. The verification provides a mathematical, not physical, compensation, in less time than other methods of verification by means of the indirect measurement of geometric errors of the machine from the linear and rotary axes. This paper presents an extensive study about the appropriateness and drawbacks of the regression function employed depending on the types of movement of the axes of any machine. In the same way, strengths and weaknesses of measurement methods and optimization techniques depending on the space available to place the measurement system are presented. These studies provide the most appropriate strategies to verify each machine tool taking into consideration its configuration and its available work space.

## 1. Introduction

The incorporation of rotation axes in multiaxis machines with three, five, or more axes increases the flexibility of these machines compared with machines with only linear axes. This enables simpler machining of complex parts, due to functional reasons in sectors such as the wind sector or by design specifications. The use of this type of machine which incorporates high added value to the machined part requires high precision machining.

Assessment of the sources of error affecting the accuracy of the machine tool (MT) can be divided into random or systematic errors. Similarly, the errors can be divided into quasistatic errors, where the errors between the workpiece and the toll are the result of the machine structure, dynamic errors caused by spindle error motion, vibration of the machine structure, and so forth. It has been observed that quasistatic errors constitute 60–70% of the total errors of a machine Barakat et al. [1]. These are divided into geometric, kinematic, and thermal errors. Geometric errors are the results of structural elements and affect the repeatability and accuracy of the machine. The direction of motion generated by joints, couplings, gears, and stiffness errors causes deformations and generates kinematic errors. Meanwhile, thermal errors are the result of temperature gradients in the structure of a machine or part, which generate dimensional changes that affect machine tool accuracy.

The different verification techniques used in order to improve the accuracy of the machine are divided into direct measurement techniques [2–9] and indirect measurement techniques of errors [10–20]. Of the direct measuring techniques, geometric verification using interferometry is more effective than the other techniques [7–9]. The application of this technique has a high accuracy in measured points used in the characterization of errors. The accuracy decreases when characterization functions are used at other points which are not used in the verification procedure. When performing an indirect measurement of errors, different volumetric verification techniques make it possible to balance the combined effect of all geometric machine tool errors that the machine has in all its work space, volumetric error. It provides a uniform compensation of errors in all the work space of the machine tool. As is the case with geometric verification, the technique and technical adequacy of use is determined by the course and structural configuration of the machine being verified. Moreover, large scale measurement systems in the industry have begun conducting large machine verification by using tracking interferometry, which is the best option for this type of machine. Tracking interferometry has distinguished itself from other techniques, reducing the amount of time and operator training needed for verification [21]. In the same way, this technique allows MT geometric error compensation in machines with open architecture controls in NC-based systems. The new control allows greater flexibility in the creation and implementation of error correction functions. In these systems, error compensation requires the development of a postprocessing module that is capable of calculating geometric errors using approximation functions for mathematical compensation instead of using numerical control machine compensation tables.

Volumetric verification, understood as an intensive identification error method for a nonlinear model, was first used in the identification of complex kinematic parameters, such as those of robots [10]. Currently, this technique is being incorporated into the machine tool sector in order to perform a characterization of its geometric errors [11–19] in less time than traditional methods. This paper presents a high precision volumetric model based on a laser tracker (LT), whereby error compensation is performed in a long range MT with two linear axes and a rotation axis. The paper thus presents a study of the adequacy of different nonlinear optimization methods, the regression functions to be used depending on the type of axis and the usable space available, and the influence of measurement noise in volumetric error reduction of the MT.

## 2. Volumetric Verification: Methodology

Machine tool geometric error analysis depends on the type and configuration of the machine [20]. The use of indirect measurement techniques using LT as a measurement system [17, 18], which can measure distance up to 50 m, is the technique that best suits long range machines. To do this, it is necessary to realise an analysis of the machine tool structure through its kinematic model.

**Figure 1:**
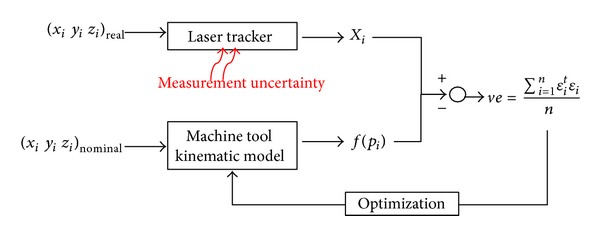
Basic parameters of volumetric verification.

Volumetric verification (Figure 1) consists of minimising the difference between real points and theoretical points introduced for numerical control (NC), through the kinematic model of the machine [21]. The differences between theoretical and real points represent the influence of combined machine errors for each point. From this, the mean square volumetric error is obtained (*ve*):

(1) $$\begin{array}{c} \varepsilon_{i}=X_{i}-f\left(p_{i}\right),\\ ve=\frac{\sum_{i=1}^{n}\varepsilon_{i}^{t}\varepsilon_{i}}{n}, \end{array}$$

where *X*_*i*_ represents the measured point coordinates of the machine tool *p*_*i*_, measured using a laser tracker, and *f*(*p*_*i*_) represents the machine tool point coordinates *p*_*i*_ obtained from the kinematic model of the machine (Figure 1).

Measurement uncertainty is affected by the noise of the measurement system, the type of machine, and the area where the MT is located. The noise measurement system is primarily determined by the LT's encoder resolution and the position measuring system to the work space of the MT. The application of techniques such as multilateration [18, 22] can decrease the measurement noise by improving the acquired data.

## 3. Machine Tool with Linear and Rotary Axes: Kinematic Model

The MT kinematic model [17, 18, 21] is used to understand and mathematically describe the motion of the machine. The sequence of movements that describes the kinematic model is determined by the type of machine, the geometrical structure, and the number of axes of the same.

**Figure 2:**
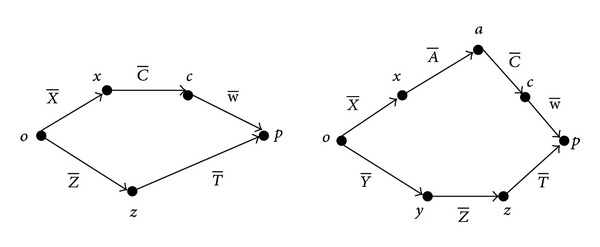
Example of kinematic scheme* XCFZ*-*XACFYZ*.

The structure of a machine is determined by the combination of different structural elements, such as guides, joints, and screws. The kinematic structure comprising these components can be modelled by a kinematic chain which symbolises the flow of movements of serial kinematic structures. The combined effect of errors is determined by the machine tool configuration being analysed. Machine types are classified according to the movement of the workpiece, the tool and axis of motion, and the linear and rotational axes of the machine tool (Figure 2).

In a kinematic scheme* XCFZ*-*XACFYZ* (Figure 2), *F*, or *o* in Figure 2, determines the fixed part of the machine. The letters to the right of *F* represent the axes that move with the tool, and letters to the left of *F* represent the axes that move with the piece. The linear axes are represented by the movement of axes *X*, *Y*, and *Z* about axes *x*, *y*, and *z*, whereas the axes of rotation are represented by the rotation of A, B, or C about axes *x*, *y*, and *z*, respectively. Similarly, *T* represents the difference (*x*, *y*, *z*) between the last axis movement of the tool and the workpiece *W* as presented by Tong et al. [22].

Assuming that a machine's motions are those of a rigid body, the motion can be implemented by a translation and rotation matrix [22–26]. Using this, the position of the tool relative to the LT system is determined as a function of the movement of the machine. The position of a tool tip relative to a measurement system in cartesian coordinates (LT) is determined by the following: the programmed nominal position, the position of the tip of the tool with respect to the reference machine (offsets), and the geometric errors of the axes.

**Figure 3:**
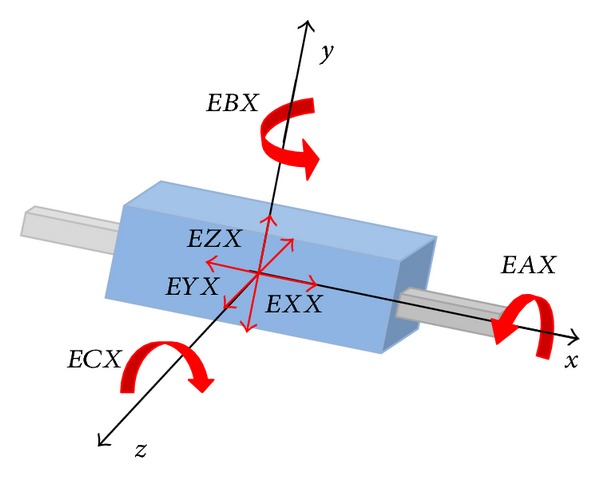
Errors of a linear axis. Movement direction *X*.

*Geometric Errors in a Linear Axis*. When facing the error analysis of a linear axis, the UNE 15300-1 is equivalent to ISO 230, which defines the errors of a linear axis (Figure 3):

 
*EXX*: linear positioning error for *x* axis,
 
*EXY*: straightness error between *y* and *x*,
 
*EXZ*: straightness error between *z* and *x*,
 
*EAX*: roll axis *x*,
 
*EBX*: yaw axis *x*,
 
*ECX*: pitch axis *k*.

**Figure 4:**
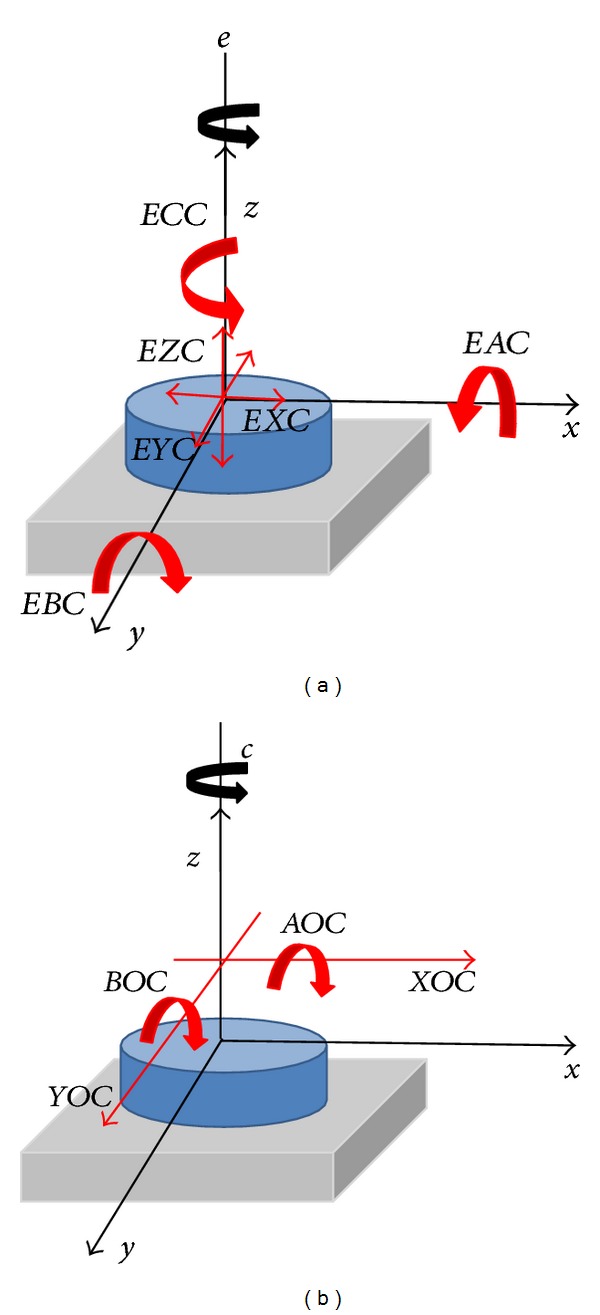
Errors on a rotational axis. Rotational axis *Z*.

*Geometric Errors in a Rotational Axis*. In tackling the analysis of errors affecting a rotation axis, the UNE-ISO 230 presents ten different errors (Figure 4) which are presented as follows:

 
*EXC*: radial movement in *X* direction,
 
*EYC*: radial movement in *Y* direction,
 
*EZC*: axial movement,
 
*EAC*: tilting movement about the *X* axis,
 
*EBC*: tilting movement about the *Y* axis,
 
*ECC*: angular position error,
 
*XOC*: position *X* of *C*,
 
*YOC*: position *Y* of *C*,
 
*AOC*: squareness of *C* on *Y*,
 
*BOC*: squareness of *C* on *X*.

**Figure 5:**
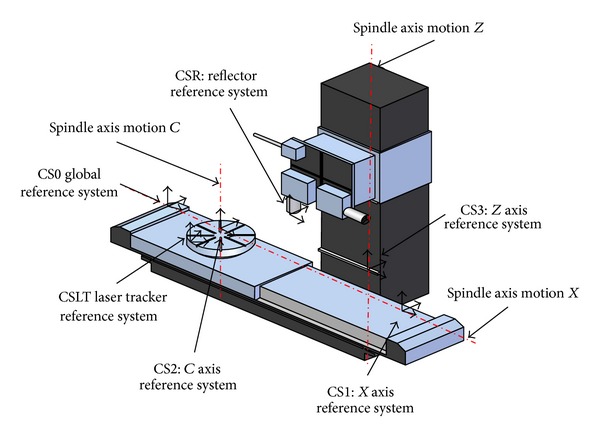
Machine tool* XCFZ* to study.

The geometric configuration of the machine on which this work has been performed corresponds to a grinding* XCFZ*. To obtain the modeling kinematic behavior, it is necessary to use 6 auxiliary coordinate systems (Figure 5):

- 1 global coordinate system (CS) CS0,
- 3 coordinate systems CS1, CS2, and CS3 associated with the axes of movement of the machine *x*, *c*, and *z*, respectively,
- 1 coordinate system associated with the tool CSR,
- 1 laser tracker coordinate system CSLT.

The kinematic scheme* XCFZ* model determined the sequence of movements of the MT from the rotation and translation matrices corresponding to each of the axes:

(2) X−+R= −1(x)C−+R= −1(x)Rg= −1(c)R= −1(c)W−=Z−+R= −1(z)T−.

$\overset{-}{T}$ represents the milling tool offset:

(3) $$\begin{array}{c} \overset{-}{T}=\left(\begin{array}{c} x_{T}\\ y_{T}\\ z_{T} \end{array}\right). \end{array}$$

$\overset{=}{R}\left(k\right)$ represents the rotation error matrix on the *k*-axis of the machine tool:

(4) $$\begin{array}{c} \overset{=}{R}\left(k\right)=\left(\begin{array}{ccc} 1 & ECk & -EBk\\ -ECk & 1 & EAk\\ EBk & -EAk & 1 \end{array}\right)\;k=X,C,Z. \end{array}$$

$\overset{=}{Rg}(c)$ represents the nominal rotation matrix on the *x* axis:

(5) $$\begin{array}{c} \overset{=}{Rg}\left(c\right)=\left(\begin{array}{ccc} \cos\left(c\right) & -\sin\left(c\right) & 0\\ \sin\left(c\right) & \cos\left(c\right) & 0\\ 0 & 0 & 1 \end{array}\right). \end{array}$$

$\overset{-}{X}$ represents the linear error vector in the *x*-axis of the milling machine:

(6) $$\begin{array}{c} \overset{-}{X}=\left(\begin{array}{c} -X+EXX\\ ECX\\ EZX \end{array}\right). \end{array}$$

$\overset{-}{C}$ represents the linear error vector in the *y*-axis of the milling machine:

(7) $$\begin{array}{c} \overset{-}{C}=\left(\begin{array}{c} EXC\\ ECC\\ EZC \end{array}\right). \end{array}$$

$\overset{-}{Z}$ represents the linear error vector in the *z*-axis of the milling machine:

(8) $$\begin{array}{c} \overset{-}{Z}=\left(\begin{array}{c} EXZ-Z\cdot EBO\\ ECZ-Z\cdot EAO\\ Z+EZZ \end{array}\right). \end{array}$$

$\overset{-}{W}$ represents the piece's coordinates.

The* XCFZ* configuration of the machine determines the placement of the measurement system. The laser tracker is placed on the table associated with the movement of the rotational axis *C*, while the reflector occupies the position reserved for the tool along the kinematic chain determined by the movement of axis *Z*.

The use of an LT as a measuring system, which works with absolute coordinates, requires its incorporation into the kinematic model of the machine. Otherwise, there is a scaled difference between the model and the measured points. Therefore, the kinematic model is incorrect because of the position and orientation of the LT system with respect to the coordinate system CS2. The measurement system is defined in the kinematic model of the machine with ${\overset{-}{X}}_{\text{LT}}$, reflector coordinates in the LT reference coordinate (SCLT), and $\overset{=}{R}\left(\text{LT}\right)$, the rotation matrix between the LT reference system and rotational reference system CS2:

(9) $$\begin{array}{c} {\overset{-}{X}}_{\text{LT}}=\left(\begin{array}{c} x_{\text{LT}}\\ y_{\text{LT}}\\ z_{\text{LT}} \end{array}\right),\\ \overset{=}{R}\left(\text{LT}\right)=\left(\begin{array}{ccc} 1 & R_{\text{LT}z} & -R_{\text{LT}y}\\ -R_{\text{LT}z} & 1 & R_{\text{LT}x}\\ R_{\text{LT}y} & -R_{\text{LT}x} & 1 \end{array}\right). \end{array}$$

In an ideal kinematic model, at P (0, 0, 0), all CSs are at the origin of the global system CS0. Therefore, all axes should be cut in space. This hypothesis is discarded due to the kinematic structure that describes the layout of the elements responsible for the movement. Therefore, it is necessary to introduce an offset between each axis ${\overset{-}{C}}_{O}$, ${\overset{-}{Z}}_{O}$, which will be considered depending on the software for control of the MT:

(10) C−O=(XOCYOCZOC),Z−O .=(XOZYOZZOZ),X−+R= −1(x)(C−O+C−)+R= −1(x)Rg= −1(c)R= −1(c)T−LT +R= −1(x)Rg= −1(c)R= −1(c)R= −1(LT)X−LT=(Z−O+Z−)+R= −1(z)T−.

Isolation of the coordinate ${\overset{-}{X}}_{\text{LT}}$ of point part P (reflector) in the LT reference system is represented as follows:

(11) X−LT=R= −1(LT)(Rg=(c)R=(c)R=(x)     ×((Z−O+Z−)+R= −1(z)T−       −X−−R= −1(x)(C−O+C−))−T−LT).

**Figure 6:**
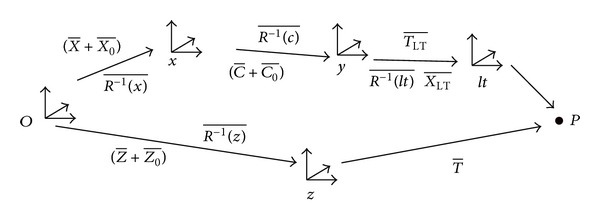
Kinematic scheme of the studied machine tool.

The kinematic model presented in Figure 6 shows that errors *XOC*, *YOC*, *AOC*, and *BOC* have not been considered directly. These errors are considered as installation errors of the turntable on the linear axis *X*. The effect of these errors is absorbed by other errors of the same axis approximation functions (see Section 5.2).

## 4. Generation of Synthetic Tests: Data Capture

One of the advantages that volumetric verification has against other verification techniques is the reduction of the time spent on data capture. To improve the time reduction, it is necessary to perform an automatic capture of points from a previously created numerical control program.

**Figure 7:**
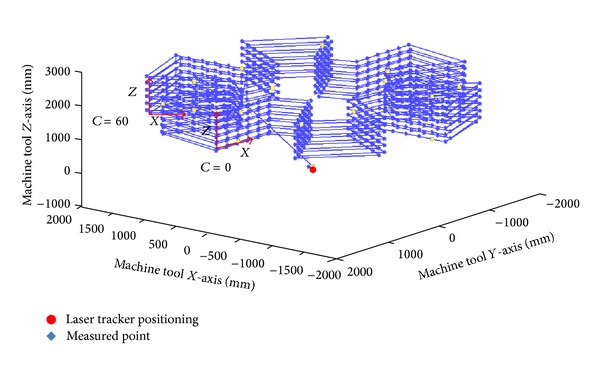
Discretization of the machine tool work space. Joint characterization of errors.

The spatial distribution of the points to be measured is determined according to the most widely used operating ranges for each machine during machining. If the machining is performed in a machine work space, a homogeneous distribution of points is used. The measured points are used to provide an overall correction giving equal weight to the entire area of work space (Figure 7). However, if machining is performed in a particular area, the spatial point distributions to be measured are centred there. This provides a better error compensation on this area compared to the other ones.

In order to characterize the different variables of influence on the identification parameter procedure, a synthetic data parametric generator is used [17, 18]. This simulates the data capture process on a particular machine using its kinematic model. Different verification procedures are studied using a synthetic test in order to obtain the greatest error reduction in the shortest time possible.

### 4.1. Parametric Synthetic Data Generator

The parametric generator consists of a set of algorithms that provide synthetic points around the workspace of the MT. Synthetic points are affected by individual kinematic errors and their combined effects on the measurement noise [17].

**Figure 8:**
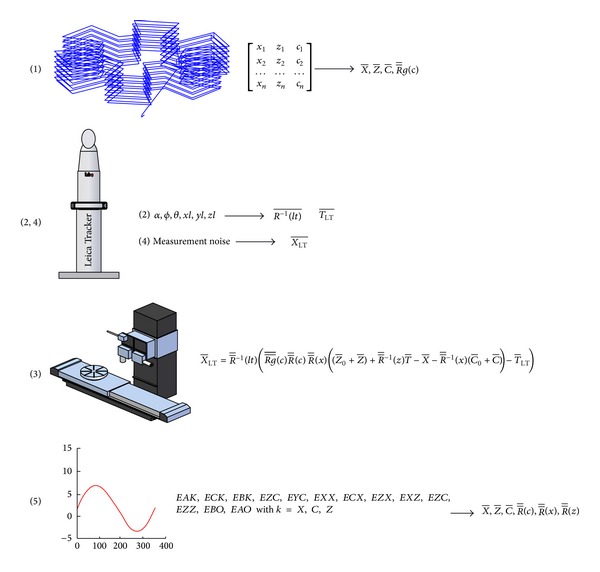
Input Parameters of synthetic test parametric generator.

The parameters required for the correct operation of the generator are similar to those needed in a real test (Section 6), together with polynomials that characterize errors presenting the MT (Figure 8):

1. nominal MT points to measure,
2. LT position and orientation with respect to the coordinate system associated with the axis of motion along which the piece is aligned,
3. kinematic model of the machine tool,
4. the characteristics of the measuring systems used and measurement noise of LTs,
5. geometric synthetic errors of each of the axes that make up the kinematic chain of the MT.

The generated synthetic test represents faithfully the real test. It has the advantage of knowing each of the factors that affect the test. Using the synthetic test, the most suitable method for planning and later volumetric verification of this kind of machine is studied.

## 5. Process and Method of Obtaining the Approximation Errors Functions

As stated in Section 2 of this paper, volumetric verification consists of minimising the difference between real points and theoretical points introduced for numerical control, through the kinematic model of the machine. The minimization of this difference is carried out through an iterative process of parameter identification based on a model of nonlinear optimization.

**Figure 9:**
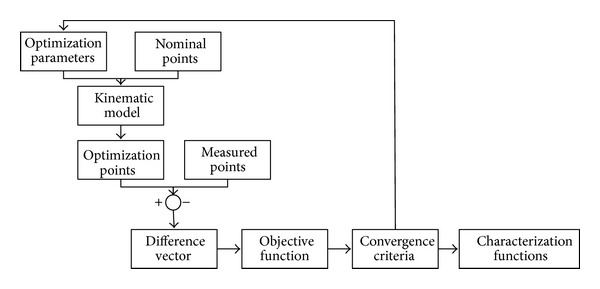
Errors characterization procedures.

From the MT kinematic model, nominal points introduced using NC in CSMT are transformed into the LT coordinates system. Once the points are in the same coordinate system, the difference between each pair of points is calculated (Figure 9). To obtain the approximation functions of each geometrical error, a series of decisions must be taken about the objective function to minimize, the convergence criteria, and the optimization parameters.

A previous paper by Aguado et al. [18] has studied the influence of different objective functions to minimize, as well as the influence of convergence criteria and the parameters to use. The objective function that provides the greatest volumetric error reduction is minimization of the mean vector of differences between the real and optimization points:

(12) Ei=(xreal,i−xopt,i)2+(yreal,i−yopt,i)2+(zreal,i−zopt,i)2,Φ=∑i=1nEin.

The minimization of the objective function Φ is performed by varying the optimization parameter vector. This vector is formed for each of the coefficients and/or parameters from which the approximation functions of each of the errors are obtained. The approximation functions depend on regression functions used in the identification procedure. The machine type to be verified and the physical behaviour of its geometrical errors determine the regression function to use.

The choice of the degree of each of the approximation functions is a factor which will have a substantial influence on the optimization. The higher the order of the polynomial, the less the residual sum of squares. If the degree employed is high, it may incorporate the influence of nonrepeatable errors like the measurement noise on the characterisation functions. Kruth et al. [26] said that geometric errors normally vary slowly along the work space of the machine tool. Therefore, a third order polynomial is suitable for error approximations.

The physical behavior of the movement axes of a machine* XCFZ* is not the same for all movements of the machine. The linear axes of the machine *X*, *Z* are characterized by Legendre, Chebyshev, or polynomial simple regression functions. Axis *C*, the rotational axis, is characterized using periodic functions as a result of the periodic behavior of their geometric errors. Therefore, the vector of parameters of identification depends on the type of the machine and the regression functions used.

### 5.1. Regression Functions for Linear Axes

Volumetric error reduction depends on polynomial regression functions $\overset{ˇ}{f}\left(x\right)$ used to characterize geometric errors. Variable *x* determines the function of dependence and not the *x* component of a point. If error to minimize belongs to *y* axis, dependence variable *x* has the *y* coordinate of measured points. The same applies to all axes and coordinates.

Simple polynomials express *f*(*x*) as $\overset{ˇ}{f}(x)$ with

(13) $$\begin{array}{c} \overset{ˇ}{f}\left(x\right)=a_{0}+a_{1}x+a_{2}x^{2}+\cdots +a_{n}x^{n}. \end{array}$$

The model identification parameters are the weights *a*_*i*_ which form each of the approximation errors functions.

If the optimization is performed using Chebyshev polynomials, the error function *f*(*x*) is characterized by

(14) $$\begin{array}{c} \overset{ˇ}{f}\left(x\right)=\overset{n}{\underset{i=0}{\sum }}\theta_{i}T_{i}\left(x\right), \end{array}$$

where *n* is the degree of the approximate function, *θ*_*i*_ the weight associated with each Chebyshev polynomial of order *i*, and *T*_*i*_(*x*) the Chebyshev polynomial of order *i*. A Chebyshev polynomial of order *i* can be defined by the following form:

(15) $$\begin{array}{c} T_{i+1}\left(x\right)=2xT_{i}\left(x\right)-T_{i-1}\left(x\right),\\ T_{o}\left(x\right)=1,\\ T_{1}\left(x\right)=x. \end{array}$$

The weights *θ*_*i*_ are obtained from Chebyshev nodes *z*_*i*_, from *m* number of points which divide the domain of the following function:

(16) $$\begin{array}{c} z_{i}=-\cos\left(\frac{\left(2i-1\right)\pi }{2m}\right),\\ \theta_{o}=\frac{1}{m}\overset{m}{\underset{i=1}{\sum }}f\left(z_{i}\right),\\ \theta_{j}=\frac{2}{m}\overset{m}{\underset{i=1}{\sum }}T_{j}\left(z_{i}\right)f\left(z_{i}\right). \end{array}$$

The value of the function nodes *f*(*z*_*i*_) is modified to obtain the approximation functions of each of the errors. The parameter has a physical meaning. It represents the value that should have the function which approximates the node *z* on m nodes that has divided the domain of error.

The use of Legendre polynomials [27–30] approximate *f*(*x*) as:

(17) $$\begin{array}{c} \overset{ˇ}{f}\left(x\right)=\overset{n}{\underset{i=0}{\sum }}a_{i}P_{i}\left(x\right),\\ P_{0}\left(x\right)=1\;\;P_{1}\left(x\right)=x,\\ \left(n+1\right)P_{n+1}=\left(2n+1\right)xP_{n}-nP_{n-1}. \end{array}$$

The optimization parameter vector will be constituted by *a*_*i*_ weights of each linear axis error of the MT. The difference between Legendre and simple polynomials lies in the dependence weights *a*_*i*_ between themselves. Therefore, the Legendre polynomials are more efficient than simple polynomials.

It should be noted that geometric errors have a physical behavior, whereas the volumetric verification provides a mathematical correction of the combined effect of all of them. Using constraints in the optimization procedure, the characterization is guided in relation to their behavior, improving the real meaning function approximation. These restrictions affect the number of parameters used in the identification process. In a linear axis, all errors should be zero for point P(0 0 0). Therefore, the independent coefficient of each approximation function used in the characterization is zero. This reduces to 12 terms the identification parameters vector, if simple polynomials are used as regression functions and 12 × *m* if the regression functions are Chebyshev or Legendre polynomials.

### 5.2. Regression Functions for Rotational Axis

In order to realise a proper volumetric verification of any machine tool with rotational axes, such as* XCFZ*, it is necessary to obtain the approximation functions of each of the errors of a rotational axis.

The physical behavior of the geometric errors of this type of axis makes it impossible to characterize them by a simple, Legendre, or Chebyshev polynomial of order three. This is due to the periodic behavior of these geometric errors. To realize a better characterization of the errors, periodic functions such as Fourier series must be used. Fourier series provide the behavior of the error in unevaluated areas from the measurement of the partial turn of the rotational axis.

**Figure 10:**
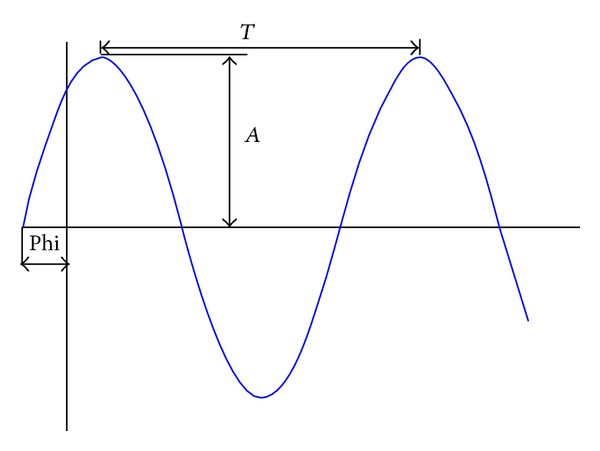
Fourier series.

An alternative is to characterize these geometric errors using Fourier series as follows:

(18) $$\begin{array}{c} \tilde{f}\left(x\right)=\overset{n}{\underset{i=1}{\sum }}A_{i}\cdot \text{sen}\left(\frac{2\pi }{T}\cdot {\theta_{i}}_{z}\right)+B_{i}\cdot \cos\left(\frac{2\pi }{T}\cdot {\theta_{i}}_{z}\right) \end{array}$$

or using series expansion of sines as follows:

(19) $$\begin{array}{c} \tilde{f}\left(x\right)=\overset{n}{\underset{i=1}{\sum }}A_{i}\cdot \text{sen}\left(\frac{2\pi }{T}\cdot {\theta_{i}}_{z}+\varphi_{i}\right), \end{array}$$

where *A*_*i*_ and *B*_*i*_ are the amplitude of the error, *T* is the period error, *θ*_*z*_ is the rotated angle in each position, and *φ*_*i*_ is the offset of the origin, as in Figure 10.

A rotational axis presents ten geometric errors according to UNE-230-2; the kinematic model presented in Section 3 only uses six errors* EXC*,* EYC*,* EZC*,* EAC*,* EBC*, and* ECC*, leaving aside the other four errors* XOC*,* YOC*,* AOC*, and* BOC*. These four errors are considered the constant result of mounting errors of the turntable about the axis of movement *X*. These errors have the effect that the normal plane of rotation does not coincide exactly with the centre of rotation* XOC* and* YOC*. In the same way, the normal of the rotational axis is not square to the coordinate system of the rotational table, producing two errors* BOC* and* AOC*. Therefore, a displacement error *D* is added in order to absorb the influence of these errors. Thus, a geometric error is defined by (20) using Fourier series or (21) using series expansion of sines:

(20) $$\begin{array}{c} \tilde{f}\left(x\right)=D+\overset{n}{\underset{i=1}{\sum }}A_{i}\cdot \text{sen}\left(\frac{2\pi }{T}\cdot {\theta_{i}}_{z}\right)+B_{i}\cdot \cos\left(\frac{2\pi }{T}\cdot {\theta_{i}}_{z}\right), \end{array}$$

(21) $$\begin{array}{c} \tilde{f}\left(x\right)=D+\overset{n}{\underset{i=1}{\sum }}A_{i}\cdot \text{sen}\left(\frac{2\pi }{T}\cdot {\theta_{i}}_{z}+\varphi_{i}\right). \end{array}$$

The optimization parameter vector is formed by the displacement of each axis error *D*, the amplitude *A*_*i*_ and the phase shift *φ*, which depends on the degree of the polynomial, the nominal coordinate *θ*_*i*__*z*_ but not of the phase *T* which is considered as a constant of value 2*π*.

### 5.3. Optimization Methods

To tackle the volumetric verification of a machine with linear and rotational axis* XCFZ*, the method of optimization is extended in comparison with the methods for machines with three linear axes [17, 18].

#### 5.3.1. Phases of Optimization

Different configurations of optimization produce different results depending on the method selected (Table 1). If different phased optimizations of identification parameters are realised, the results will clearly differ depending on the strategy employed.

**Figure 11:**
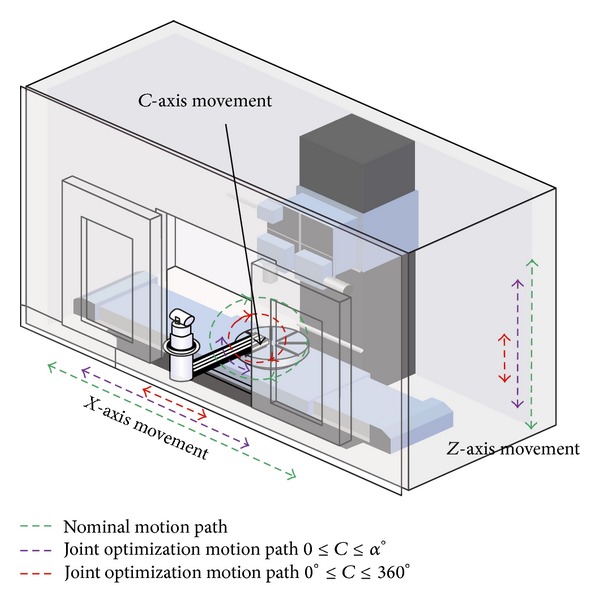
Range of movement in relation to the characterization method.

The application of this technique is directly related to the path of each of the machine axes to verify. Note that the LT measurement system in a machine tool with* XCFZ* configuration must be placed on the turntable (Figure 11).

Figure 11 shows a representation of a real machine. As can be seen, the trail of the* xcz* axes is much larger than the path that can be measured by the LT as a result of the LT being placed on the turntable and rotated to different positions of the *C*-axis.

In order to realise the characterization of all geometric errors of a machine using this method, regardless of the number of stages used, it is necessary to place the LT on a profile attached to the turntable. Consequently, the LT is available to measure the range of axes *x* and *z* in different positions of *c* between 0° and 360°. This measurement method is limited, either by protective housings of the machine or by its structure, and therefore the range of the axes that can be measured depends on the method selected.

In order to realise a verification in which the largest range of each axis can be certificated, different methods with their scopes and limitations are presented.

#### 5.3.2. Independent Optimization of Linear and Rotational Axes

**Figure 12:**
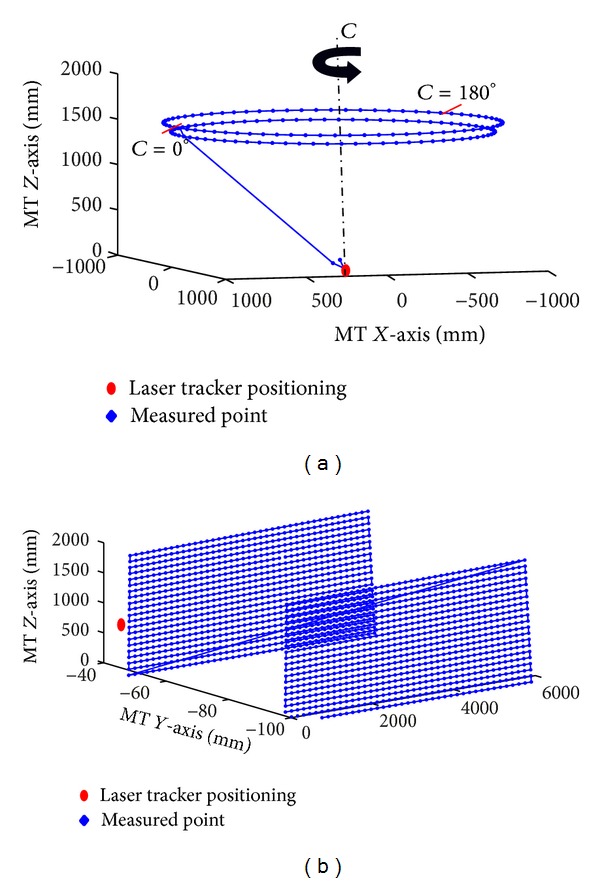
Volume discretization to an independent analysis of the errors.

This method studies the effect of each set of errors depending on the type of motion which each axis provides (Figure 12). It is necessary to perform an independent parameter identification of the errors from the linear and rotation axes.

In a machine tool* XCFZ*, the errors from the *XZ* plane formed by the linear axis movements and the errors of the rotational axis of the turntable should be independently analyzed (Figure 12). If this technique is employed, it achieves a longer range of measurement in the *XZ* plane as well as *C*. Looking at the example of Figure 11, this method makes it possible to analyze around the *C* axis range labelled with a red line and the range of *X* and *Z* marked in purple.

The realization of this characterization methodology has the disadvantage of losing a lot of points. This is because the plane *XZ* is generated with a long range of axes. This mesh cannot be repeated in different positions of *C*. Similarly, the effect of *C* axis geometric errors is known only for a few points with *XZ* movements, due to the new *XZ* mesh having to be measured in all positions of *C*.

#### 5.3.3. Combined Optimization of Linear and Rotational Axes

**Figure 13:**
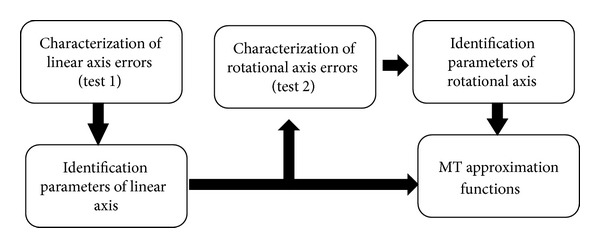
Scheme of optimization for a combined characterization of linear and rotational axes.

With the aim of increasing the number of points of the rotation mesh, a plane of *XZ* points is generated which can be measured in different positions on the LT when the turntable turns around *C* (Figure 13). In this test, only errors in rotation axis *C* are characterized. However, when crossing a mesh *XZ* these are also affected by the errors of linear axes *X* and *Z*. This test characterizes only the errors of the rotational axis. However, when the mesh of points of the plane *XZ* is measured, its points are affected by errors of linear axes *X* and *Z*.

In order to determine the influence of the errors of linear axes in the *XZ* plane points, a prior characterization of the linear axis error Test 1 is required (Figure 13). The characterization of these errors is made from a mesh with a long range of *X* and *Z* for *C* = 0°. Once the approximation functions of the linear axes are obtained, their identification parameters are introduced in the optimization parameter vector from which the rotation axis errors in Test 2 are obtained (Figure 13).

It is desirable for the plane of points *XZ* used to characterize the errors of linear axes and the plane of points *XZ* used to characterize the rotation axis errors to have common points. Similarly, it is desirable for the plane of point *XZ* used to characterize the errors of linear axes with *C* = 0° also to be measured for other values of *C*. Thus, both tests have points in common and the approximation functions have been obtained with points that have considered the combined effect of all errors.

The different configurations of the optimization presented different results depending on the method selected. So we present a study of the different methods in Section 6.

## 6. Procedure and Tests

In order to show the scope of the verification method presented in this paper, the results of a series of synthetic tests created using the synthetic data parametric generator are presented [17, 18].

The arrangement of the LT measuring system is similar to the global coordinate system of the machine. The machine offsets and the reflectors' offsets used are presented in Tables 2 and 3. Both are considered constant and known throughout all tests.

The use of two offsets aims to provide a greater physical meaning of the approximation functions obtained [18], preventing any coupling between the various possible errors of each of the axes.

In relation to errors introduced in the kinematic model, the combined influences of all of them are presented both as mean and maximum values. These parameters are the mean error in module (EMM) and the maximum error in module (EMax). In order to simplify the direct comparison between the initial volumetric error and the volumetric error after compensation, the residual error is calculated. This is the percent difference between the final EMM and initial EMM.

### 6.1. Common Optimization Parameters to All Tests

The EMM final value is not only affected by the optimization method used but also by the convergence criteria and constraints used on optimization.

Variation of the model parameters identification (Section 3) from which the objective function is calculated continues until one of the convergence criteria is met:

1. minimum variation on identification parameters,
2. maximum allowable variation of the objective function between two consecutive iterations,
3. number of iterations,
4. maximum permitted operations.

In order to select those convergence criteria with which the smallest residual error is obtained at the lowest possible computational cost, several studies were realised [18].

**Figure 14:**
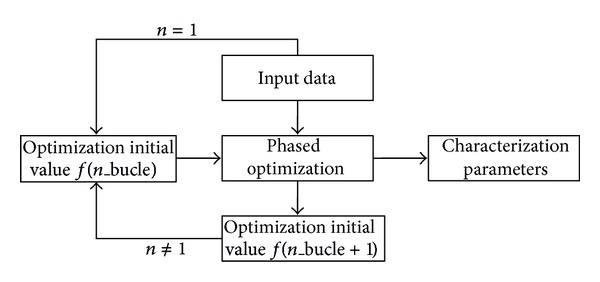
Optimization with subloops.

The best result is obtained by performing a loop optimization objective function. Each subloop has its own optimization criteria. Global optimization is formed by as many subloops (independent optimizations) as desired (Figure 14).

**Figure 15:**
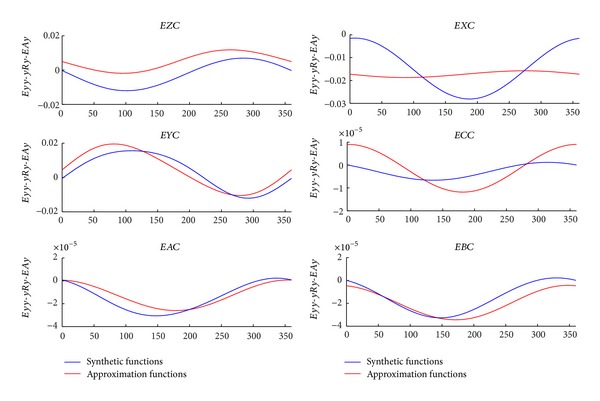
Comparison between generation functions and approximation functions obtained.

The computational cost reduction is directly related to the size of the optimization vector used. Using constraints in the optimization [18] not only achieves reduced computational cost but also gives physical meaning to the approximation functions obtained. However, the volumetric verification is a mathematical compensation, not a physical one. Volumetric verification provides a compensation of the combined effect of all errors and not the effect of each one independently (Figure 15).

The restrictions are mainly based on the suppression of the independent coefficient of the linear axis approximations functions and the suppression of the period coefficient in rotational axis errors. They are based also on suppression of the squareness between the linear axes using the linear component of the straightness error of each linear axis to calculate them. It was presented by Aguado et al. [18].

### 6.2. Results

Using the parametric synthetic data generator presented in Section 4.1, an extensive study of the different verification methods of a machine tool with configuration* XCFZ* is realised (Figure 11).

Firstly, the suitability of the periodic regression functions used in the characterization of a rotational axis is studied. To do that, the test of Table 4 is realised.

The result of Table 5 shows how the use of the sines series improved on the characterization by the Fourier series to perform the characterization of all axis errors. However, when an independent characterization of each one of its geometric errors is realized, the difference between the two methods is not so great. In the tests, a strong dependence between the initial optimization parameters and the residual errors was observed.

Once the regression function used in rotation axis characterization has been selected, we study the influence of the optimization technique as well as the initial and final error. In order to know the suitability of each method of optimization, the optimization of two different tests from the following techniques was carried out:

- joint characterization of linear and rotational errors of the rotational axis,
- characterization first of linear errors and then of rotation errors of the rotational axis (TR),
- characterization first of rotational errors and then of linear errors of the rotational axis (RT).

From Table 6, it can be seen that the most appropriate method is the optimization in two phases.

The best characterization method for a rotational axis does not have to coincide with the best method optimization of the errors of the linear axes. Therefore, a study of the same method applied to the mesh of points *XZ* was realised (Table 7).

Table 8 presents the results of optimization by characterization of errors in one phase using Chebyshev polynomials as the most appropriate optimization method for linear axes *XZ* of the machine* XCFZ*.

After studying the different optimization techniques, the influence of measurement noise was then studied in two different tests. Measurement noise was characterized by a normal distribution of value (±30 *μ*rad, ±30 *μ*rad, and ±4 *μ*m ±0.8 *μ*m/m) for spherical coordinates (azimuth, polar, and radial).

Tables 9 and 10 show how the measurement noise affects the error reduction achieved through the independent analysis of errors of each type of axis, keeping the introduced geometric errors and the convergence criteria.

A decrease in the influence of the measurement noise on the volumetric error of the machine tool can be realised through the use of the technique of multilateration. For this, it is necessary to take into consideration both multilateration techniques as the LT self-calibration techniques [23].

When the functions regression to use for each type of axis and the optimization method are known, the influence of the measurement method in relation to the space available for measurement is studied:

 Test 1: mesh formed by plane *XZ* points at intervals of 100 mm and 75 mm, respectively, for *C* = 0 as shown in Table 7,
 Test 2: mesh formed by the fixed point *XZ* according to Table 4,
 Test 3: *XZ* plane at intervals of 300 mm and 225 mm starting and finishing according to Table 7 with 0° ≤ *C* ≤ 360° at intervals of 45°,
 Test 4: *XZ* plane at intervals of 200 mm and 150 mm starting and finishing according to Table 7 with 0° ≤ *C* ≤ 360° at intervals of 30°,
 Test 5: same measurement as Test 3 using errors of linear axes from Test 1.

In Table 11, Tests 1 and 2 shows the results of an independent characterization of the axes of movement according to its type. Test 3 shows the results of the joint verification errors of all axes. Test 5 shows the result of rotary axis characterization once the errors of linear axes have been characterized in Test 1 and compensated. Test 4 is taken as a reference test by covering the whole volume of the MT.

The appropriateness of extrapolating the approximation functions obtained in each one of the tests to the rest of the MT workspace is shown in Table 12. For this, geometric errors characterized by the different methods were used to compensate a reference test, Test 4.

The independent optimization of error in relation to the type of axes in two different tests, Test 1 and Test 2, produces a strong increase of the residual error of the reference test. Therefore, these methods are unsuitable (Table 12). The approximation functions obtained from a smaller mesh size *XZ* at different positions of *C*, Test 3 (Table 11), provide better error compensation in the reference test. Similarly, the combined optimization of geometrical errors through an independent optimization of the errors of the linear axes, Test 5, provides a better compensation than any other method in the reference test. However, this correction is less than the joint optimization of all errors in the reference test. Therefore, this method is especially useful when it is not possible to realise a complete measurement of all machine tool workspace or when the number of points to measure is too high.

The behavior of the machine and the value of the errors before and after compensation can be observed using coloured maps and vector diagrams.

**Figure 16:**
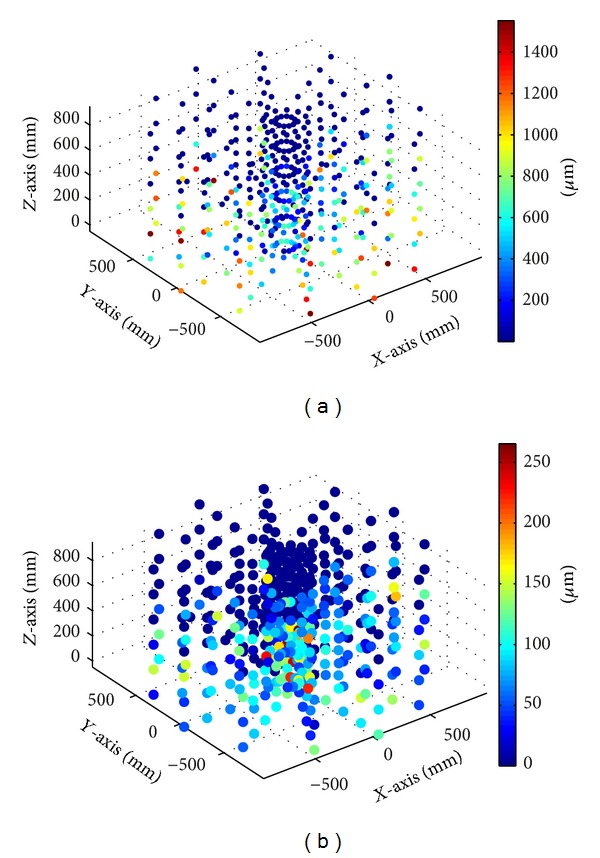
Uncompensated errors (a). Compensated errors (b). Colour maps.

A coloured map (Figure 16) provides information on the error reduction at each of the points of the workspace. Homogeneous error compensation is observed (Figure 16).

**Figure 17:**
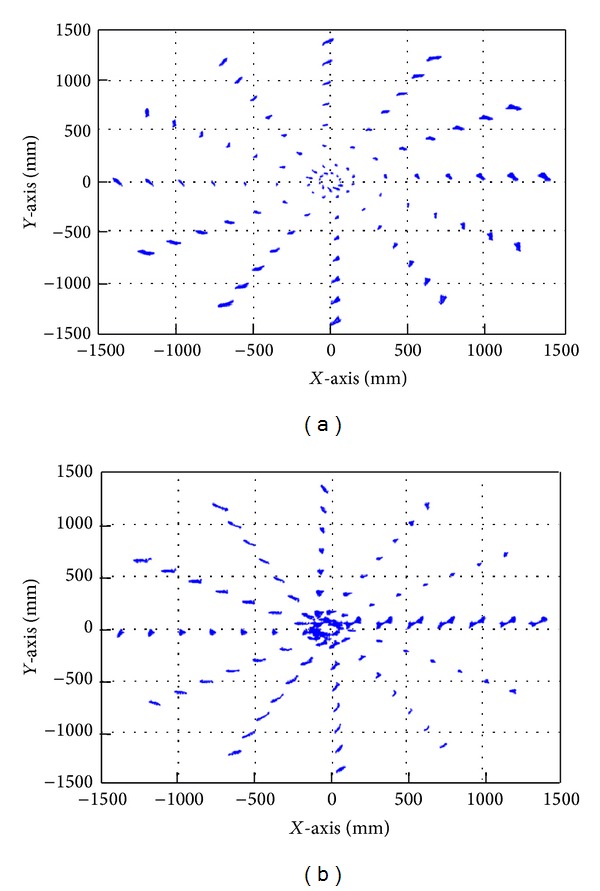
Uncompensated errors (a). Compensated errors (b). Vector diagrams.

Similarly, the vector diagram (Figure 17) shows how the physical behaviour of the machine tool errors before compensation is reduced, providing a mathematical, not physical, compensation.

## 7. Conclusions

The scope of the global volumetric error reduction depends on optimization convergence criteria, the optimization method employed, the regression functions used, and the uncertainty introduced by the measurement system and the MT.

The regression functions used depend on the type of axis of motion. The linear axes are characterized by simple, Chebyshev, or Legendre polynomials, while errors of the axes of rotation are obtained by Fourier series or sines series. Characterization by the Chebyshev polynomial produced better results than simple and Legendre polynomials. However, the use of a high number of Chebyshev nodes increases the computational cost. In relation to the periodic functions of the rotational axis, the characterization using sines series provides a greater reduction than Fourier series.

The characterization of geometric errors gives different results depending on the optimization sequence used. In linear axis characterization, the joint characterization of all geometric errors provides a residual error of 0.9% versus 2.7% with a TR optimization or 5.4% using an RT optimization. In the case of a rotational axis, the best result is obtained by performing an optimization in two phases, with 9.6% of residual error if the RT method is employed or 7.8% when the TR is used. However, if the method selected is optimization with only one phase, the residual error is 57.7% of the initial one.

The availability of space in the verification limits the number and distribution of points used in the verification. A combined optimization of the errors from rotational and linear axes provides the greatest reduction in error, if it is not available to realise a measurement of points in all the MT workspace. This method provides a residual error of 6.90% instead of 5.83%. The combined optimization improved the result of a joint characterization of a minor workspace from which a residual error of 10.14% is obtained instead of 5.83%.

## Figures and Tables

**Table 1:** Optimization with phases.

Optimization	Square	Translation	Rotation
1 phase	1	1	1
2 phases	1	2	2
3 phases rotation-translation	1	3	2
3 phases translation-rotation	1	2	3

1→ optimization of the parameters associated with each type of error in the first phase.

2→ optimization of the parameters associated with each type of error in the second phase.

3→ optimization of the parameters associated with each type of error in the third phase.

**Table 2:** Position reflector relative to SC3.

	*X* (mm)	*Y* (mm)	*Z* (mm)
Reflector 1	−50	50	−50
Reflector 2	−10	100	50

**Table 3:** Offset MT.

	*X* (mm)	*Y* (mm)	*Z* (mm)
*X*-axis	0	0	0
*Y*-axis	0	0	0
*Z*-axis	1000	25	1500

**Table 4:** Discretization of the work space of the rotational axis.

	Initial	Final	Interval
*X*-axis (mm)	0	0	0
*C*-axis (°)	0	360	5
*Z*-axis (mm)	0	0	0

**Table 5:** Fourier series versus sines series.

	EMM initial (*μ*m)	E.Max initial (*μ*m)	EMM final (*μ*m)	E.Max final (*μ*m)	Residual error
Fourier series	181.15	500.25	93.28	227.78	51.49%
Sines series	181.15	500.25	16.58	60.72	9.15%

EMM: mean error in module, E.Max: maximum error.

**Table 6:** Results of rotational axis verification in *XCFZ* machine.

Average initial error in module (*μ*m)	**22.7**	**194.9**
Maximum initial error (*μ*m)	**37.6**	**213.5**

Average final error in module 1 phase (*μ*m)	14.4	112.6
Maximum final error 1 phase (*μ*m)	19.1	186.1
Residual error 1 phase (%)	**63.6**	**57.7**

Average final error module 2 phases TR (*μ*m)	2.7	15.1
Maximum final error 2 phases TR (*μ*m)	4.2	65.1
Residual error 2 phases TR (%)	**11.9 **	**7.8**

Average final error module 2 phases RT (*μ*m)	1.3	18.7
Maximum final error 2 phases RT (*μ*m)	3.1	64.8
Residual error 2 phases RT (%)	**5.9**	**9.6**

**Table 7:** Discretization of the work space to linear axis of *XCFZ* MT.

	Initial	Final	Interval
*X*-axis (mm)	0	1400	100
*C*-axis (°)	0	0	0
*Z*-axis (mm)	0	600	50

**Table 8:** Linear axes characterization.

	Simple	Legendre	Chebyshev (5 nodes)
Average initial error in module (*μ*m)	737.1	737.1	737.1
Maximum initial error (*μ*m)	2010.6	2010.6	2010.6

Average final error in module 1 phase (*μ*m)	6.6	3.6	4.4
Maximum final error 1 phase (*μ*m)	23.2	10.46	16.7
Residual error 1 phase (%)	**0.9**	**0.5**	**0.1**

Average final error module 2 phases TR (*μ*m)	19.9	75.18	57.1
Maximum final error 2 phases TR (*μ*m)	36.6	244.3	1578
Residual error 2 phases TR (%)	**2.7**	**10.2**	**7.7**

Average final error module 2 phases RT (*μ*m)	39.6	123.8	8.4
Maximum final error 2 phases RT (*μ*m)	150.6	374.0	19.0
Residual error 2 phases RT (%)	**5.4**	**16.8**	**1.2**

**Table 9:** Rotational axis characterization with and without measurement noise.

Average initial error in module (*μ*m)	**230.5**	**227.0**
Maximum initial error (*μ*m)	**407.2**	**375.8**

Average final error in module (*μ*m)	30.8	16.6
Maximum final error (*μ*m)	65.7	29.7
Residual error (%)	**13.3**	**7.36**
Average noise error (*μ*m)	31.3	
Maximum noise error (*μ*m)	57.8	

**Table 10:** Linear axis characterization with and without measurement noise.

Average initial error in module (*μ*m)	**740.0**	**737.1**
Maximum initial error (*μ*m)	**2092.2**	**2010.6**

Average final error in module (*μ*m)	51.2	6.6
Maximum final error (*μ*m)	120.2	23.6
Residual error (%)	**6.9**	**0.9**
Average noise error (*μ*m)	46.8	
Maximum noise error (*μ*m)	104.8	

**Table 11:** Optimization results depending on the method used.

	A. initial	Max. initial	A. optimization	M. optimization	Residual
	E.M. (*μ*m)	error (*μ*m)	E.M. (*μ*m)	error (*μ*m)	error %
Test 1	582.48	1654.03	26.02	87.59	4.47%
Test 2	194.91	213.46	15.1	65.06	7.75%
Test 3	555.84	1523.42	51.76	191.13	9.31%
Test 4	628.69	1893.27	36.64	104.81	5.83%
Test 5	555.84	1523.42	30.24	75.06	5.44%

A.E.M. → average error in module.

Max., M. → maximum.

**Table 12:** Optimization compensation results in a reference test depending on the optimization method used.

	A. initial	Max. initial	A. optimization	M. optimization	Residual
	E.M. (*μ*m)	error (*μ*m)	E.M. (*μ*m)	error (*μ*m)	error %
Test 1	628.69	1893.27	224.54	662.32	35.72%
Test 2	628.69	1893.27	58000	141000	9225.53%
Test 1 + 2	628.69	1893.27	58000	141000	9225.53%
Test 3	628.69	1893.27	69.04	278.31	10.98%
Test 4	628.69	1893.27	36.64	104.81	5.83%
Test 5	628.69	1893.27	43.41	149.84	6.90%
